# Oxcarbazepine for refractory epilepsy: systematic review of the literature

**DOI:** 10.1590/S1516-31802009000300008

**Published:** 2009-10-06

**Authors:** Humberto Saconato, Gilmar Fernandes do Prado, Maria Eduarda dos Santos Puga, Álvaro Nagib Atallah

**Affiliations:** 1 MD, PhD. Adjunct professor in the Universidade Federal do Rio Grande do Norte (UFRN), Natal, Rio Grande do Norte, Brazil.; 2 MD, PhD. Professor in the Discipline of Emergency Medicine and Evidence-Based Medicine, Department of Medicine, Universidade Federal de São Paulo (Unifesp), São Paulo, Brazil.; 3 MHS. Affiliated researcher at Brazilian Cochrane Center and postgraduate student in the Discipline of Emergency Medicine and Evidence-Based Medicine, Department of Medicine, Universidade Federal de São Paulo (Unifesp), São Paulo, Brazil.; 4 MD, PhD. Full professor of the Discipline of Emergency Medicine and Evidence-Based Medicine, Department of Medicine, Universidade Federal de São Paulo, Escola Paulista de Medicina (Unifesp-EPM), São Paulo, Brazil.

**Keywords:** Anticonvulsants, Epilepsy, Review [Publication Type], Seizures, Oxcarbazepine [Substance name], Anticonvulsivos, Epilepsia, Revisão [Tipo de Publicação], Convulsões, Carbamazepina

## Abstract

**CONTEXT AND OBJECTIVE:**

It has been estimated that 50 million people worldwide suffer from epilepsy and around 30% will not achieve adequate control over the disease. The aim was to evaluate the effectiveness of oxcarbazepine for refractory partial or generalized epilepsy.

**METHODS:**

Systematic review. A search was conducted in the PubMed, Lilacs, EMBASE and CENTRAL databases. Studies were analyzed using the Cochrane Collaboration methodology.

**RESULTS:**

Four randomized clinical trials of medium to poor methodological quality were included. Among the adult patients, the chances that they would obtain a 50% reduction in seizure frequency were greater after using oxcarbazepine at doses of 600 mg (relative risk, RR 2.11; 95% confidence interval, CI 1.32 to 3.35), 1,200 mg (RR 3.24; 95% CI 2.11 to 4.98) and 2,400 mg (RR 3.83; 95% CI 2.59 to 5.97). Among the children, the response in the group using oxcarbazepine was also greater (RR 2.11; 95% CI 1.32 to 3.35). The oxcarbazepine doses of 1,200 mg (RR 17.59; 95% CI 2.37 to 130.35) and 2,400 mg (RR 25.41; 95% CI 6.26 to 103.10) were effective for keeping patients probably free from seizures, but the dose of 600 mg was not. There was no significant difference between oxcarbazepine and carbamazepine for controlling the crises.

**CONCLUSIONS:**

There is moderate evidence indicating that oxcarbazepine is effective as an alternative treatment for partial or generalized epilepsy in children and adults who were refractory to previous treatment.

## RESUMO

**CONTEXTO E OBJETIVO:**

Estima-se que 50 milhões de pessoas no mundo sofrem de epilepsia e cerca de 30% não obterão controle adequado da doença. O objetivo foi de avaliar a efetividade de oxcarbazepina na epilepsia parcial ou generalizada refratária.

**MÉTODOS:**

Revisão sistemática. A busca foi nas bases de dados PubMed, Lilacs, EMBASE e CENTRAL. Os estudos foram analisados segundo a metodologia da Cochrane Colaboration.

**RESULTADOS:**

Foram incluídos quatro ensaios clínicos aleatórios de média a má qualidade. Entre os pacientes adultos as chances de obterem redução de 50% na frequência de convulsões foram maiores após uso de oxcarbazepina na dose de 600 mg (risco relativo, RR 2.11; intervalo de confiança, IC 95% 1,32 a 3,35; na dose de 1.200 mg (RR 3,24; IC 95% 2,11 a 4,98) e na dose de 2.400 mg (RR 3,83; IC 95% 2,59 a 5,97). Entre as crianças a resposta no grupo usando oxcarbazepina também foi significativamente maior (RR 2,11; IC 95% 1,32 a 3,35). Oxcarbazepina mostrou probabilidade dos pacientes ficarem livre de convulsões, ser eficaz nas doses de 1.200 mg (RR 17,59; IC 95% 2.37 a 130,35) e 2.400 mg (RR 25,41; IC 95% 6,26 a 103,10) não foi eficaz na dose de 600 mg. Não houve diferença estatística significante entre oxcarbazepina e carbamazepina no controle das crises.

**CONCLUSÕES:**

Há evidências moderada de que a oxcarbazepina é um tratamento eficaz como alternativa para os casos de epilepsia parcial ou generalizada em crianças e adultos que tenham sido refratários a tratamento prévio.

## INTRODUCTION

Epilepsy is a disease characterized by a wide range of symptoms resulting from a variety of cerebral disorders. Semiologically, it is classified as partial or generalized. The clinical manifestations can arise through sensitive, sensory, psychological, vegetative and motor signs and symptoms of simple or complex nature, depending on the neural system implicated in the genesis of the disease. Epilepsy crises have a recurrent nature and tend to always present with the same characteristics over long periods. Epilepsy is not a single disease but represents a variety of diseases with underlying cerebral dysfunctions that may have different causes. Thus, a priori, it comprises a heterogenous clinical entity.^1^

It has been estimated that 50 million people worldwide suffer from epilepsy. Partial epilepsy is the most common form, occurring in around 60% of epilepsy patients. Up to 30% of epilepsy patients will not achieve adequate control over the disease.^2^

It is important to differentiate convulsions from epileptic seizures. Convulsions signify transitory occurrences of signs and symptoms resulting from synchronic or excessively abnormal neuronal activity in the brain, triggered by convulsogenic factors. These include metabolic disorders involving glucose, electrolytes, increased temperature or cranial-encephalic trauma, among others. On the other hand, epilepsy is a cerebral disorder with long-lasting predisposition towards generating epileptic crises, which lead to neurobiological, cognitive, psychological and social consequences. Status epilepticus is a severe form of clinical presentation of epileptic crises and is characterized by long duration. Depending on the type of crisis, it may have high morbidity-mortality.^1^

Antiepileptic drugs are the initial treatment for the great majority of epilepsy patients. For 150 years, physicians have been prescribing antiepileptic drugs to patients with recent diagnoses of epilepsy, without any formal scientific evaluation regarding the efficacy, safety and tolerability of such drugs. For example, phenobarbital and phenytoin were registered and put on the market without any randomized clinical trial having been conducted to evaluate their efficacy and safety.^3^ Indication of a specific drug for a particular condition of epilepsy is based on clinical studies with varying levels of evidence, many without sufficient methodological quality. Drugs are chosen with regard not only to the data on the clinical trials available, but also to variables such as the type of epilepsy, the patient’s age and the supposed mechanism of action of the drug.

The action of antiepileptic drugs occurs through several mechanisms. In the case of oxcarbazepine (a carbamazepine derivative), the action is through selective blockade of sodium channels. Oxcarbazepine is almost completely absorbed after a single oral dose and is rapidly metabolized to the active monohydroxy form, which is the major pharmacologically active component of this substance.^4^^,^^5^

Oxcarbazepine is considered effective for treating partial epilepsy in adults and children, both as monotherapy and in association with other antiepileptic drugs.^5^ When used as monotherapy, it is recommended that carbamazepine [oxcarbamazepine] should be started at a dose of 600 mg per day, divided into two doses. If necessary, the dose can be increased every week until achieving control over the seizures or until reaching a maximum dose of 2,400 mg per day.^5^

In patients with recently diagnosed epilepsy, the therapeutic response is commonly observed at a dosage of 1,200 mg per day. In cases that are refractory to monotherapy, the therapeutic response is most frequently achieved at a dosage of 2,400 mg per day.^5^

## OBJECTIVES

The objectives of the present review were: to evaluate whether oxcarbazepine is effective and safe for treating refractory epilepsy; whether oxcarbazepine used as monotherapy is effective and safe for treating refractory epilepsy in adults and children; and whether the use of oxcarbazepine as adjuvant treatment is effective and safe for treating refractory epilepsy in adults and children.

## METHODS

### Data sources and searches

A wide-ranging search was conducted in several electronic databases in order to identify all the relevant randomized clinical trials that have evaluated the effectiveness of oxcarbazepine for treating refractory epilepsy.

There were no language restrictions. Studies were considered eligible whether published or not.

The following sources and strategies were used in searching for studies:

### Electronic databases

#### Cochrane Central Register of Controlled Trials (CENTRAL; Cochrane Library Vol. 2, 2008)

Phase 1 - Oxcarbazepine
Phase 2 - “Epilepsy OR Epilepsia OR Seizure”
Phase 3 - #1 AND #2

#### Medical Literature Analysis and Retrieval System Online (Medline), via PubMed interface (1966 - July 2008)

#1 (“oxcarbazepine “[Substance Name]) OR (oxcarbazepine) OR (GP 47680) OR (Timox) OR (Desitin brand of oxcarbazepine) OR (Trileptal) OR (Novartis brand of oxcarbazepine)
#2 (“Epilepsy”[Mesh]) OR (Epilepsy) OR (Epilepsies) OR (Epileptic Seizures) OR (Epileptic Seizure) OR (Seizure, Epileptic) OR (Seizure Disorder) OR (Seizure Disorders) OR (Seizures, Epileptic) OR (Single Seizure) OR (Seizure, Single) OR (Seizures, Single) OR (Single Seizures) OR (Aura) OR (Auras) OR (Awakening Epilepsy) OR (Epilepsy, Awakening) OR (Epilepsy, Cryptogenic) OR (Cryptogenic Epilepsies) OR (Cryptogenic Epilepsy) OR (Epilepsies, Cryptogenic) OR (REFRACTORY EPILEPSY)
#3 (randomized controlled trial [pt] OR controlled clinical trial [pt] OR randomized controlled trials [mh] OR random allocation [mh] OR double-blind method [mh] OR single-blind method [mh] OR clinical trial [pt] OR clinical trials [mh] OR (“clinical trial” [tw]) OR ((singl* [tw] OR doubl* [tw] OR trebl* [tw] OR tripl* [tw]) AND (mask* [tw] OR blind* [tw])) OR ( placebos [mh] OR placebo* [tw] OR random* [tw] OR research design [mh:noexp] OR comparative study [mh] OR evaluation studies [mh] OR follow-up studies [mh] OR prospective studies [mh] OR control* [tw] OR prospectiv* [tw] OR volunteer* [tw]) NOT (animals [mh] NOT humans [mh])
#4 = #1 AND #2 AND #3

#### Literatura Latino-Americana e do Caribe em Ciências da Saúde (Lilacs)

(oxcarbazepine) OR (oxcarbazepine) OR (GP 47680) OR (Timox) OR (Desitin brand of oxcarbazepine) OR (Trileptal) OR (Novartis brand of oxcarbazepine) [Palavras] and (Epilepsy) OR (Epilepsies) OR (Epileptic Seizures) OR (Epileptic Seizure) OR (Seizure, Epileptic) OR (Seizure Disorder) OR (Seizure Disorders) OR (Seizures, Epileptic) OR (Single Seizure) OR (Seizure, Single) OR (Seizures, Single) OR (Single Seizures) OR (Aura) OR (Auras) OR (Awakening Epilepsy) OR (Epilepsy, Awakening) OR (Epilepsy, Cryptogenic) OR (Cryptogenic Epilepsies) OR (Cryptogenic Epilepsy) OR (Epilepsies, Cryptogenic) OR (REFRACTORY EPILEPSY) [Palavras]

#### Excerpta Medica Database (Embase)

Phase 1 - Oxcarbazepine
Phase 2 - “Epilepsy OR Epilepsia OR Seizure”
Phase 3 - #1 AND #2

#### Websites

Websites of clinical trial registers were investigated in order to find any possible randomized clinical trials in progress.

http://www.controlledtrials.com

http://clinicaltrials.gov/ct/gui

#### Lists of references

The lists of references of the clinical trials identified and references in review articles were scrutinized to locate any additional studies that were not located through the databases.

## CRITERIA FOR STUDY INCLUSION

### Types of studies

Randomized or quasi-randomized trials.

### Types of participants

Patients with any type of refractory partial or generalized epilepsy were included. Cases in which the patients continued to present seizures despite using at least two antiepileptic drugs were classified as refractory epilepsy. Recently diagnosed epilepsy patients without previous treatment were not included.

### Types of intervention

The types of intervention included were oxcarbazepine versus placebo, oxcarbazepine versus other antiepileptic drugs and oxcarbazepine as adjuvant treatment for another antiepileptic drug versus other antiepileptic drugs.

Randomized clinical trials comparing different dosages of oxcarbazepine that did not have a control group, taking placebo or another drug, were not included.

### Types of outcomes

- Reduction in seizure frequency of at least 50%: proportion of patients with a reduction in seizure frequency of 50% or more over the treatment period, compared with the baseline pre-randomization period.
- Absence of convulsive crises during the follow-up period.
- Treatment dropout rate: the proportion of the patients that abandoned the treatment because of its lack of efficacy or because of adverse events.
- Adverse events.

This study could not evaluate cognitive and quality-of-life effects, because no randomized clinical study evaluating these outcomes was identified.

The titles and abstracts of all the articles were scrutinized. Complete photocopies of all relevant studies or studies that fulfilled the inclusion criteria were obtained. After reading the articles, the studies that fulfilled the inclusion criteria were evaluated with regard to methodological quality, and all the relevant information was extracted from them.

To qualitatively evaluate the methodology of the randomized clinical trials, the criteria described by Schulz et al. and the criteria described in the Handbook of the Cochrane Collaboration were used.^7^

Methodological quality was not used as an exclusion criterion.

### Data-gathering methods

A standard form was used for extracting the relevant information from each article that was included.

### Data analysis

For dichotomous variables, relative risk and risk difference with 95% confidence intervals were calculated by means of fixed models. For continuous variables, the weighted mean difference and respective confidence interval were used.

The RevMan 5 statistical package supplied by the Cochrane Collaboration was used to perform meta-analysis. If meta-analysis could not be performed, the study results were converted into relative risks or weight mean differences with their respective confidence intervals.

In the event of a meta-analysis with statistically significant heterogeneity (P < 0.1), the randomized model was adopted. For comparisons in which statistical significance was observed, the number needed to treat or number needed to harm was calculated.

The presence of heterogeneity was investigated by means of the chi-squared test and the I^2^ test. The heterogeneity was considered statistically significant when I^2^ was greater than 50% and the P-value was less than < 0.10 (< 10%). Values of I^2^ between 30% and 50% were taken to suggest notable but nonsignificant heterogeneity.^7^

## RESULTS

Four randomized clinical trials were identified. Three of them compared oxcarbazepine with placebo and one of them compared oxcarbazepine with carbamazepine among patients with difficult-to-control epilepsy (Tables 1 and 2).^8^^,^^9^^,^^10^^,^^11^^,^^12^^,^^13^^,^^14^^,^^15^^,^^16^^,^^17^^,^^18^

Characteristics of the studies included

The study by Schachter included patients from the age of 12 years upwards who had presented two to ten epileptic crises, of which at least one was a complex partial seizure that had not responded to treatment. The patients were randomly distributed to receive oxcarbazepine 2,400 mg or placebo.^19^ Among the outcomes planned for the present systematic review, the study by Schachter included only the incidence of seizure-free patients and the incidence of adverse effects. The follow-up period for assessing the treatment efficacy was only 10 days in length.

The study by Barcs included men and women between the ages of 15 and 65 years with simple or complex crises of partial or generalized type, in accordance with the classification of the International League Against Epilepsy (1981 and 1989), which were not adequately controlled using one to three antiepileptic drugs concomitantly.^20^ The patients randomly received oxcarbazepine 600 mg, or oxcarbazepine 1,200 mg, or oxcarbazepine 2,400 mg or placebo. The outcomes analyzed were: 50% reduction in seizure frequency, incidence of seizure-free patients, dropout due to adverse events, incidence of adverse events and adverse events classified as serious. The follow-up period was only 29 days in length.^20^

The study by Glauser included children between the ages of three and seventeen years with simple or complex crises of partial or generalized type, in accordance with the classification of the International League Against Epilepsy (1981 and 1989), which were not adequately controlled using one or two antiepileptic drugs concomitantly.^21^ Patients who fulfilled the inclusion criteria were randomly distributed to receive oxcarbazepine 30 to 46 mg/kg or placebo. The outcomes analyzed were: incidence of seizure-free patients, dropout due to adverse events, incidence of adverse events and adverse events classified as serious. The follow-up period was 112 days.^21^

The study by Reinikainen included adult epilepsy patients with an unsatisfactory response to phenytoin or patients who had presented undesirable effects with this drug. The patients were randomized to receive oxcarbazepine 200 mg, increasing gradually to 800 mg; or carbamazepine 300 mg, increasing to 1,200 mg. The outcomes analyzed were: seizure frequency, seizure-free patients and incidence of side effects. The follow-up period was three months.^22^

**Table 1.** Results from searching through the literature in the databases

Database	Citations	Studies identified
PubMed	195	4
Embase	138	4
CENTRAL	129	4

**Table 2.** Characteristics of the studies excluded

Study	Reasons for exclusion
Christe et al. (1997)^8^	This was a clinical trial evaluating the effect of oxcarbazepine on recently diagnosed epilepsy patients, without previous treatment, and therefore it did not include refractory patients.
Guerreiro et al. (1997)^9^	This was a clinical trial evaluating the effect of oxcarbazepine on recently diagnosed epilepsy patients, without previous treatment, and therefore it did not include refractory patients.
Kutluay et al. (2003)^10^	This was not a randomized clinical trial.
Passarella et al. (2005)^11^	This was not a randomized clinical trial.
Albani et al. (2004)^12^	This was a randomized clinical trial comparing different strategies for replacing carbamazepine with oxcarbazepine, and there was no comparison with placebo or other drugs.
Albani et al. (2007)^13^	This was not a randomized clinical trial.
Beydoun et al. (2000)^14^	This was a randomized clinical trial comparing different dosages of oxcarbazepine, and there was no comparison with placebo or other drugs.
Beydoun et al. (2003)^15^	This was not a randomized clinical trial.
Bill et al. (1997)^16^	This was a clinical trial evaluating the effect of oxcarbazepine on recently diagnosed epilepsy patients, without previous treatment, and therefore it did not include refractory patients.
Piña-Garza et al. (2005)^17^	This was a randomized clinical trial comparing different dosages of oxcarbazepine, and there was no comparison with placebo or other drugs.
Sachdeo et al. (2001)^18^	This was a randomized clinical trial comparing different dosages of oxcarbazepine, and there was no comparison with placebo or other drugs.

### Evaluation of methodological quality

**Figure 1.**
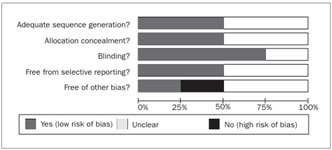
Bar chart showing the risk of bias from combining the studies that were included. It can be seen that there was a moderate to high risk of bias.

The four studies included could be classified as presenting poor to moderate quality. A brief description of the methodological characteristics is given below, and the risk of bias can be seen in Figure 1.

### Generation of allocation sequence

The studies by Barcs and Reinikainen^20^^,^^22^ did not describe the method used for generating allocations. In the studies by Schachter and Glauser, a computer was used to generate the randomization sequence.^19^^,^^21^

### Allocation concealment

The studies by Barcs and Reinikainen^20^^,^^22^ did not describe the method used for concealing the allocations. The study by Schachter reported that the code was kept closed, but the method was not described. Central randomization was used in the study by Glauser.^19^^,^^21^

### Blinding

All of the studies were described as double-blind, but there was no information about which aspects involved were blinded. The placebo was described as identical in the studies by Glauser et al.^21^ and Schachter et al.^19^ In the study by Reinikainen et al., which compared oxcarbazepine with carbamazepine, the two tablets that were offered were described as identical.^22^

### Outcome results

### Oxcarbazepine versus placebo

#### *50% reduction in seizure frequency (* *Figure 2* *)*

Two studies analyzed the outcome “60% reduction in seizure frequency”. The study by Barcs included adult patients, while the study by Glauser only included children. Both among the adults and among the children, oxcarbazepine was shown to be statistically more effective than placebo, independent of the dose. An increase in the effect could be seen with increasing dosage.^20^^,^^21^ Among the adult patients, the response rate after using oxcarbazepine at a dose of 600 mg was 27% (45/168) (risk relative, RR 2.11; 95% confidence interval, CI 1.32 to 3.35; number needed to treat, NNT = 7); at a dose of 1,200 mg, it was 41% (73/177) (RR 3.24; 95% CI 2.11 to 4.98; NNT = 4); and at a dose of 2,400 mg, it was 50% (87/174) (RR 3.83; 95% CI 2.59 to 5.97; NNT = 3); while in the placebo group, the response rate was 13% (22/173). Among the children, the response rate in the group using oxcarbazepine was 41% (57/138), while in the placebo group it was 22% (28/129) (RR 2.11; 95% CI 1.32 to 3.35; NNT = 5).

**Figure 2.**
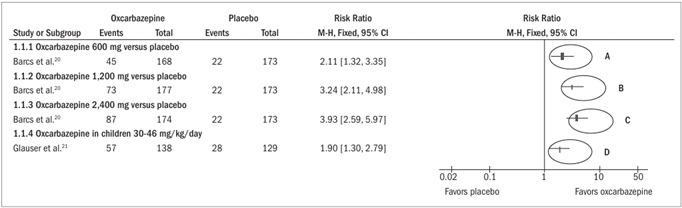
Forest plot showing that oxcarbazepine was more effective than placebo for reducing the frequency of crises by 50%, independent of the dosage or whether it was given to adults or children, because the horizontal line representing the confidence interval for each comparison did not cross the vertical line representing relative risk of 1.0, at any of the dosages analyzed (ellipses A, B, C and D).

#### *Patients without seizures during the follow-up period (* *Figure 3* *)*

It was possible to perform a meta-analysis on the comparison between oxcarbazepine 2,400 mg and placebo, from the studies by Schachter et al.^19^ and Barcs et al.^20^ At this dose, the rate of patients who were seizure-free with the use of oxcarbazepine was 23% (51/225), while in the placebo group, the rate was 1% (2/224). This difference was statistically significant (RR 25.41; 95% CI 6.26 to 103.10) and resulted in a risk difference of 22% and an NNT of 5, thus signifying that it would be necessary to treat five patients with oxcarbazepine for there to be one seizure-free patient. This meta-analysis did not show heterogeneity.

At the dosages of 600 mg and 1,200 mg, only the study by Barcs et al. was included.^20^ Oxcarbazepine at a dose of 600 mg was not shown to be more effective than placebo (RR 5.15; 95% CI 0.61 to 43.61), whereas at the dosage of 1,200 mg, a statistically significant benefit was observed. The response rate after using the drug was 10% (18/177), compared with 1% among the patients who received placebo (RR 17.59; 95% CI 2.37 to 130.35).

Among children, oxcarbazepine was not statistically different from placebo when the outcome was to put a complete end to the crises (RR 4.67; 95% CI 0.55 to 39.47).

**Figure 3.**
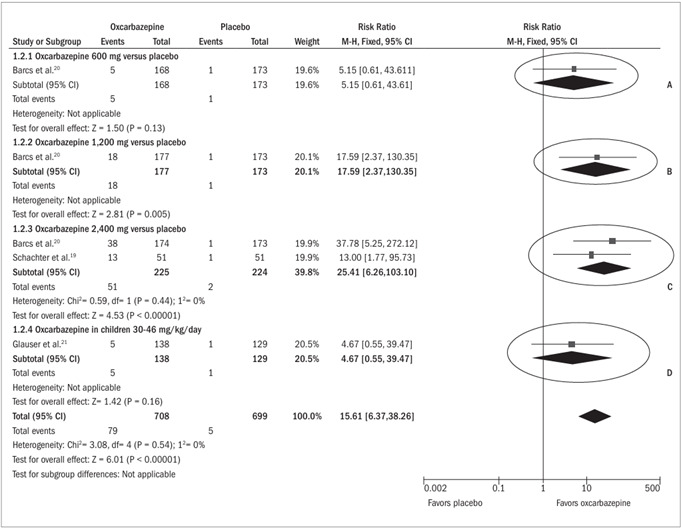
Forest plot showing that oxcarbazepine was more effective than placebo with regard to leaving the patients free from crises, for adult patients at the dosages of 1,200 mg (ellipse B) and 2,400 mg (ellipse C), because the horizontal line representing the confidence interval for each comparison did not cross the vertical line representing relative risk of 1.0. At the dosage of 600 mg (ellipse A) and among children (ellipse D), oxcarbazepine was no more effective than placebo because the confidence interval included the relative risk of 1.0.

#### *Dropout due to adverse events (* *Figure 4* *)*

The studies by Barcs et al.^20^ and Glauser et al.^21^ were included. No meta-analysis was performed. Among the adult patients, the dose of 600 mg did not present any increase in the risk of dropout due to adverse events (RR 1.37; 95% CI 0.73 to 2.59), but at the doses of 1,200 mg and 2,400 mg, the dropout rates were respectively 36% (64/177) and 67% (116/174) and were statistically significant in relation to placebo, which had a rate of 9% (15/173).

Among children using oxcarbazepine, the dropout rate due to adverse events was 10% (14/138), while among those using placebo, it was 3% (4/129). This difference was statistically significant (RR 3.27; 95% CI 1.11 to 9.68).

**Figure 4.**
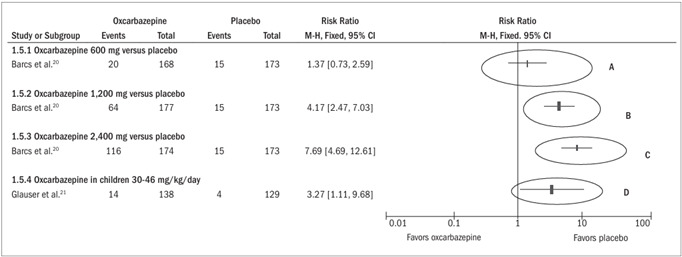
Forest plot showing that the risk of dropping out from the treatment because of adverse events was statistically greater among the adult patients who used oxcarbazepine at the dosages of 1,200 mg (ellipse B) and 2,400 mg (ellipse C), and among children (ellipse D), because the horizontal line representing the confidence interval for each comparison did not cross the vertical line representing relative risk of 1.0. However, at the dosage of 600 mg (ellipse A), the chance of dropping out because of the use of oxcarbazepine was no greater, because the confidence interval included the relative risk of 1.0.

#### *Incidence of adverse events (* *Figure 5* *)*

It was possible to perform meta-analysis in relation to the dose of 2,400 mg because two studies (Schachter et al.^19^ and Barcs et al.^20^) could be grouped. At this dosage, the rate of adverse events among patients receiving oxcarbazepine was 91% (208/228), compared with 72% in the placebo group. This difference was statistically significant (RR 1.27; 95% CI 1.16 to 1.39).^19^^,^^20^ Five patients needed to be treated with oxcarbazepine for there to be one adverse event more than with placebo.

At the dose of 600 mg, no statistically significant difference in the risk that adverse events would occur was observed (RR 1.1; 95% CI 0.99 to 1.22). With the use of oxcarbazepine at the dose of 1,200 mg, an increase in the rate of adverse events in relation to placebo was observed (90% versus 76%). This difference was statistically significant (RR 1.18; 95% CI 1.08 to 1.30). Thus, for every seven patients treated, there would be one adverse event more than with placebo.

Among children, no statistically significant difference in the risk of adverse events was observed (RR 1.04; 95% CI 0.87 to 1.24).

**Figure 5.**
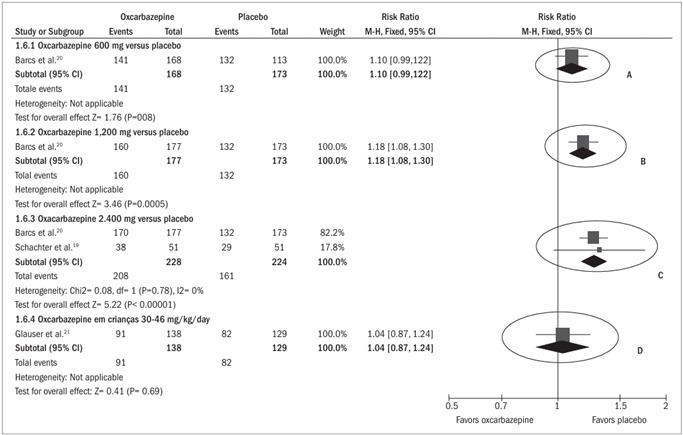
Forest plot showing that the risk of adverse events was statistically greater among the adult patients who used oxcarbazepine at the dosages of 1,200 mg (ellipse B) and 2,400 mg (ellipse C), because the horizontal line representing the confidence interval for each comparison did not cross the vertical line representing relative risk of 1.0. Thus, the chances of presenting adverse events after dosages of 1,200 mg and 2,400 mg were respectively 1.18 and 1.27 times greater than what was observed among the patients who used placebo. However, among the adults who used the dosage of 600 mg (ellipse A) and among the children (ellipse D), the chance that adverse events might occur because of the use of oxcarbazepine was no greater, because the confidence interval included the relative risk of 1.0.

#### *Incidence of adverse events classified as serious (* *Figure 6* *)*

Only the study by Barcs et al. evaluated this outcome.^20^ There was no statistically significant difference in the risk that adverse events classified as serious would occur, at the doses of 600 mg (RR 1.37; 95% CI 0.59 to 3.17), 1,200 mg (RR 0.98; 95% CI 0.40 to 2.40) or 2,400 mg (RR 1.99; 95% CI 0.92 to 4.30).

**Figure 6.**
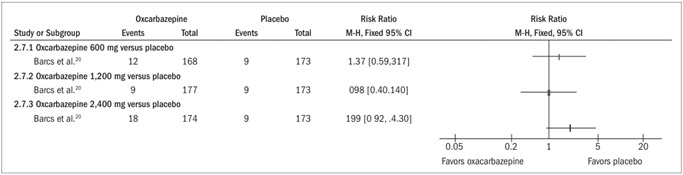
Forest plot showing that the risk of adverse events classified as serious occurring through the use of oxcarbazepine, among adult patients, was not statistically greater than through the use of placebo, independent of the dosage, because the confidence interval included the relative risk of 1.0. The study by Glauser et al.,21 which evaluated the effect of oxcarbazepine among children, did not evaluate this outcome.

#### Oxcarbazepine versus carbamazepine

**Figure 7.**
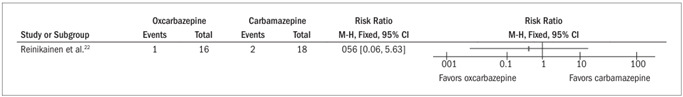
Forest plot showing that the risk that crises might worsen was not statistically different between oxcarbazepine and carbamazepine, because the confidence interval included the relative risk of 1.0.

**Figure 8.**
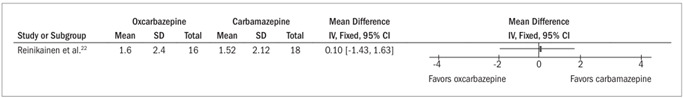
Forest plot showing that the frequency of crises was not statistically different between oxcarbazepine and carbamazepine, because the confidence interval included the relative risk of zero.

**Figure 9.**
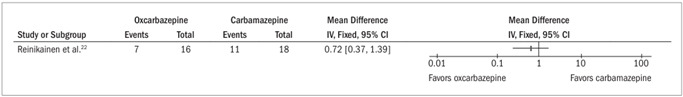
Forest plot showing that the risk of adverse events was not statistically different between oxcarbazepine and carbamazepine, because the confidence interval included the relative risk of 1.0.

Only one study comparing these two interventions was identified. No statistically significant differences in the following outcomes were observed: worsening of seizures (RR 0.56; 95% CI 0.06 to 5.63; Figure 7), seizure frequency (weighted mean difference, WMD 0.10; 95% CI -1.43 to 1.63; Figure 8) or incidence of adverse events (RR 0.96; 95% CI 0.37 to 1.39; Figure 9).

## DISCUSSION

After an exhaustive search in several databases with the aim of proving whether oxcarbazepine was effective and safe for treating refractory epilepsy, we only identified four randomized clinical trials. Three clinical trials compared oxcarbazepine with placebo and one other clinical trial compared oxcarbazepine with carbamazepine. No other clinical trials comparing oxcarbazepine with other antiepileptic drugs were identified. Meta-analyses could only be performed in relation to certain variables for the comparison between oxcarbazepine at the dose of 2,400 mg and placebo.

Among adults, it can be affirmed that oxcarbazepine is effective for treating refractory epilepsy, with regard to obtaining a 50% reduction in the frequency of crises. The best results were obtained at the dosages of 1,200 mg and 2,400 mg, since at these dosages, the patients’ chances of remaining free from crises were greater than at the dose of 600 mg. This response profile demonstrates that there is a dose-response gradient, with greater efficacy as the dose is increased. However, the dosage of 2,400 mg presented lower tolerance, thus resulting in a much higher dropout rate due to adverse events, such that 67% of the patients using this dose dropped out of the treatment because of adverse reactions, compared with a dropout rate of 36% among those who received 1,200 mg.

With regard to the outcome of putting a complete end to the crises, even under conditions in which oxcarbazepine was more effective than placebo, the rate of patients without crises was very small, although greater than what was observed with placebo (1%). For example, at the dose of 1,200 mg, only 10% presented this response in a study with only 28 days of follow-up. At a dose of 2,400 mg, two studies with follow-ups of 10 and 28 days could be grouped in a meta-analysis, which showed that only 23% of the patients remained free from seizures.

Only one randomized study on children was identified.^21^ This used dosages of 30 to 46 mg/kg and it only showed a benefit in relation to reducing the seizure frequency by 50%. Use of the drug was not associated with an increase in the rate of seizure-free patients.^21^ Regarding safety, we observed a greater dropout rate due to adverse events, with drug use in relation to placebo.

Although non-randomized studies have shown that oxcarbazepine is better tolerated, in terms of presenting fewer adverse events than seen with carbamazepine and phenytoin, we were unable to confirm this information through studies with greater scientific robustness. Only one study comparing oxcarbazepine with carbamazepine was included, with a very small sample and dubious methodological quality. From this, no difference in efficacy and safety between these two drugs could be identified in relation to patients with refractory epilepsy.

One limitation in interpreting the results from this systematic review came from the lack of standardization of the therapeutic response between the studies, which made it difficult to perform meta-analyses. Another important limitation was the very short follow-up in some studies. In one study, the efficacy was evaluated only ten days after the treatment (Schachter et al.). The study with the longest follow-up was the one by Glauser et al., in which the response to treatment was evaluated after 112 days, i.e. just over three months.^19^^,^^21^

Ethical problems strongly influence the planning of clinical trials involving epileptic patients. It is unthinkable to leave patients with refractory epilepsy using placebo for long periods. On the other hand, it needs to be asked whether the patients who remained free from seizures over periods of 10, 28 or 112 days following their treatment, as seen in the studies included in this systematic review, would have remained free from seizures over periods of six or twelve months or even longer. The answer to this question has to be that it is unlikely and, for this reason, a minimum follow-up period of six months for evaluating the therapeutic response of antiepileptic drugs is recommended.^23^^,^^24^^,^^25^

Another matter to be considered in clinical trials is the type of outcome that would best reflect the efficacy of treatments with antiepileptic drugs. The best way of measuring the effect of such medications is through the rate of seizure-free patients because this outcome is not only unequivocal but also associated with better quality of life,^23^^,^^26^^,^^27^ in comparison with patients with a 50% reduction in seizure frequency. Nonetheless, it must always be taken into account that, for ethical reasons, it is necessary to make comparisons with other active treatments, which could consist of other drugs or treatments involving a controlled diet, for example.

## CONCLUSIONS

### Implications for practice

- There is moderate evidence indicating that oxcarbazepine is an effective alternative treatment for cases of partial or generalized epilepsy among children or adults that had been refractory to previous treatments with antiepileptic drugs.
- The evidence is insufficient to affirm whether oxcarbazepine is safe.
- No evidence regarding any effect from oxcarbazepine on cognition could be obtained.
- There is insufficient evidence to affirm that oxcarbazepine is equal or superior to carbamazepine for treating refractory epilepsy.

### Implications for research

- New randomized clinical trials evaluating the efficacy and safety of oxcarbazepine among adults and children with refractory epilepsy are needed. These new studies should have adequate randomization methods, samples with sufficient numbers of participants and follow-up periods of at least six months.
- Because of the small number of randomized clinical trials, a systematic review of observational studies is needed in order to evaluate the safety and cognitive effects of oxcarbazepine.
- Randomized clinical trials as described in the preceding paragraph, comparing oxcarbazepine with other antiepileptic drugs and with other types of clinical treatment, for treating refractory epilepsy, are needed.
